# IUC24396-89 The fate of the hydronephrotic kidney in muscle-invasive bladder cancer

**DOI:** 10.1093/oncolo/oyaf248.023

**Published:** 2025-08-29

**Authors:** M Gami, T Rich, H Motiwala

**Affiliations:** Southend University Hospital, Essex, United Kingdom; Southend University Hospital, Essex, United Kingdom; Southend University Hospital, Essex, United Kingdom

## Abstract

**Background:**

Hydronephrosis is an independent predictor of poor clinical outcomes in muscle-invasive bladder cancer (MIBC), associated with higher rates of extravesical disease and reduced survival. However, its impact on renal function remains unclear. This study evaluates changes in serum creatinine over 2 years and assesses whether de-obstruction at diagnosis influences renal function.

**Methods:**

We conducted a retrospective analysis of patients diagnosed with MIBC and hydronephrosis (2020-2024). Inclusion criteria were ≥T2 disease and serum creatinine measurements at diagnosis and 2 years post-diagnosis. Patients without oncologic intervention, those who died within 1 year, or decompression after initial treatment were excluded. Data were extracted from hospital records.

**Results:**

Among 52 patients (mean age 68.5, 30 males), most had T2/T3 disease; 19 had nodal involvement (N+), and 11 had metastases (M+). Treatment included cystectomy (*n* = 25), chemoradiotherapy (*n* = 23), radiotherapy (*n* = 10), and chemotherapy alone (*n* = 4). Creatinine increased by a mean of 15 µmol/L (*P* = .0325) at one year in all treatment groups, but there was no significant difference at 2 years. In cystectomy patients, creatinine increased significantly at one year (13 µmol/L, *P* = .04) but not at 2 years. This was primarily driven by patients who were de-obstructed before cystectomy (Cr + 29.80 µmol/L, *P* = .01). Non-surgical treatments showed no impact on renal function. Most de-obstruction was pre-chemoradiotherapy.

**Conclusions:**

Patient selection for de-obstruction is critical, particularly in those receiving chemotherapy, as there is evidence that renal function can be preserved. Our findings suggest de-obstruction can be avoided when cystectomy is planned, as it may help preserve renal function at 1 year.

